# Personality Disorder and Adolescents—Still Living on a Shoestring?

**DOI:** 10.3390/children10101611

**Published:** 2023-09-27

**Authors:** Sune Bo, Majse Lind

**Affiliations:** 1 Department of Psychology, University of Copenhagen, 1353 Copenhagen, Denmark; 2 Department of Psychology, University of Aalborg, 9000 Aalborg, Denmark; majseli89@gmail.com

Personality disorder (PD) has been and continues to be a controversial mental disorder to discuss with young people under the age of 18. Despite strong actions and courageous intentions to bring PD in young people to the attention of the mental health authorities globally, the vast majority of young people with personality pathology do not receive adequate treatment. Misconceptions persist, and many clinicians regard people with PD as ‘troublesome’, ‘untreatable’, and with a life-long problematic outlook on the future. Some clinicians even believe that treating patients with PD is less important than treating patients with other mental health disorders [1].

Interestingly, we know that young people *have* a personality that seems to be structured very much like the one we find in adults (i.e., the Five Factor Model). Research also displays approximately the same personality stability in adolescents compared to people in late adulthood, and instruments developed to assess personality and personality pathology show the same acceptable psychometric properties. PD in adolescents displays moderate stability over short as well as longer periods—the same results that have been uncovered for PD in adulthood. We can assess PD in young people reliably and down to the age of 12.

PD features in adolescents predict social, academic, and vocational impairments in adulthood stronger than ‘Axis-I’ symptomatology (i.e., mood, conduct, and anxiety disorders), and 60% of adolescents with BPD report about suicide ideations and between 50 and 60% self-harm. ‘Burden of Disease’ related to PD in adolescents exceeds those found in adults with PD, and compared to young people with ‘Axis-I’ disorders, adolescents with PD exhibit more academic problems, have fewer friends, display more behavioral problems, use more alcohol, drugs, and nicotine, engage in more unsafe sexual behavior, use more medication, and experience more life crises. Specifically, BPD features in adolescents are reported as risk factors for the development of psychosis, manic episodes, depression, and impaired social functioning later in life. Additionally, BPD features in young people are related to academic failure, a less successful career, and increased health costs, already at the age of 20—impairments that seem to persist at least for two decades. Quality of life ratings are significantly reduced in young people and lower than in young people with cancer. Parents of children with PD report more stress and negative family experiences compared to parents of children with first-episode psychosis. Although there is compelling evidence that evidence-based treatment approaches are effective in reducing PD symptoms, young people with PD and their families struggle to be taken seriously and experience stronger stigma and marginalization compared with patients with other severe forms of psychopathology, and young people with PD are rarely offered the appropriate treatment. PD in adolescents has been treated as the black sheep of the ICD and DSM families, and among many clinicians, PD in young people has been surrounded by negativity, psychotherapeutic pessimism, and conceptual controversy. Thus, the significant impairment in general and social functioning may be a result of stigma and the refusal to diagnose PD early on in life and offer the right treatment, which further increases the stigmatization of this group [2].

Furthermore, we fail with respect to PD in adolescents, since we know from research on early intervention for a broad range of mental disorders in young people that early intervention and tailored, personalized treatment work. The earlier we detect and focus on mental disorders, the more effective the result and the cheaper the intervention. Treatment in the later stages of disorders is often less effective and costs more. This is also the case for PD in young people; thus, we need a clinical staging model as our interventional framework, with timely and tailored treatment offers matched to the specific stage of the personality disorder for the young person. With the introduction of ICD-11, a new dimensional approach to conceptualizing personality pathology has finally been introduced, defining the core features of PD to include self- and interpersonal dysfunctions, and the questionable specific PD categories known from ICD-10 (and DSM-5 Section II) have been abolished (except for borderline PD). With this new approach, a window of opportunity emerges to take PD in young people seriously and implement a stepped-care approach, adjusting treatment to the level of disorder severity. It is now clearly emphasized in ICD-11 that there is no minor age limit for the diagnosis of PD, and ICD-11 provides specific information about developmental presentations, i.e., how symptom presentation differs by age, to facilitate a more reliable assessment. Furthermore, ICD-11 also offers a section describing boundaries to normality, i.e., information intended to help the clinician distinguish between the disorder, subclinical symptoms, and variations in normal functioning. Hopefully, this approach will pave the way for a re-introduction of assessment and treatment of personality pathology in the group of young people under 18 years, including enhanced research activity in this field. 

This Special Issue focuses on the new dimensional approach to PD introduced in ICD-11 and what that means for young people under the age of 18 years. Methodologically, Mazreku and colleagues validated the state-of-the-art ICD-11 PD adolescent assessment in the form of the Levels of Personality Functioning Questionnaire Parent Report, while Bach and Vestergaard provided expert guidance on the diagnostic similarities and differences related to ICD-11 PD and autism spectrum disorder. 

Clinically, Akin and colleagues showed that adolescents with a wide range of mental health problems showed disturbances within ICD-11-related personality structure and conflicts, and Sharp and Cervantes demonstrated that personality functioning explained variation in personality pathology over and above general psychiatric severity in adolescents. 

Therapeutically, Simonsen and colleagues emphasized the benefits of applying short-term mentalization-based therapy for adolescents with mild to moderate PD, and Lind and colleagues explored the features of and changes in the highly overlooked construct of narrative identity in mentalization-based group therapy for adolescents with borderline PD. We are confident that this Special Issue will contribute to pushing the PD field forward in meaningful ways. Enjoy!
